# Prevalence of High Frequency Noise-Induced Hearing Loss Among Medical Students Using Personalized Listening Devices

**DOI:** 10.3390/jcm14010049

**Published:** 2024-12-26

**Authors:** Aishwarya Gajendran, Gayathri Devi Rajendiran, Aishwarya Prateep, Harshith Satindra, Rashmika Rajendran

**Affiliations:** 1 Department of Otorhinolaryngology, Head and Neck Surgery, Pinderfields Hospital, Wakefield WF1 4DG, UK; 2 Department of ENT, ACS Medical College and Hospital, Chennai 600077, Tamil Nadu, India; gayathri.r1994@gmail.com; 3 Department of Otorhinolaryngology, Head and Neck Surgery, Government Omandurar Medical College, Chennai 600002, Tamil Nadu, India; draishwaryaprateep@gmail.com; 4 SRM Medical College, Chennai 603203, Tamil Nadu, India; harshithsat@gmail.com; 5 Department of Otorhinolaryngology, Head and Neck Surgery, Bhaarath Medical College, Chennai 600073, Tamil Nadu, India; rashmikarajendran@gmail.com

**Keywords:** noise-induced hearing loss, personalized listening devices, high-frequency audiometry, medical students, hearing loss prevention

## Abstract

The misuse of personalized listening devices (PLDs) resulting in noise-induced hearing loss (NIHL) has become a public health concern, especially among youths, including medical students. The occupational use of PLDs that produce high-intensity sounds amplifies the danger of cochlear deterioration and high-frequency NIHL especially when used in noisy environments. This study aims to evaluate the incidence and trends of NIHL among medical students using PLDs. **Background/Objectives**: The purpose of this study is to assess the prevalence of high-frequency NIHL among PLD-using medical students. **Methods**: A semi-structured questionnaire covering details on PLD usage, exposure to noisy environments, and hearing difficulties was used to gather the data required. Conventional pure-tone audiometry with extended high-frequency audiometry was preceded by routine clinical evaluation using tuning fork tests and otoscopic examination for hearing loss assessment and to rule out middle-ear pathology. Hearing impairment was determined and categorized according to the Goodman and Clark classification system (250 Hz to 8000 kHz). SPSS version 21 was used in the analysis of the frequency data collected. **Results**: Out of 100 participants, using conventional PTA, 33% were found to have hearing loss, with 42.9% of males and 23.5% of females affected. Bilateral hearing loss was seen in 36.4% of the cases. Left-sided hearing loss was found to be more common (28%). The duration of usage of PLD had a significant correlation with hearing loss with a *p*-value < 0.0001. Hearing thresholds were significantly elevated at 16 kHz and 18 kHz in both the right and left ear. **Conclusions**: The high prevalence of PLD misuse among medical students is a major risk factor for NIHL. To help combat chronic hearing loss, students need to be educated about safe listening levels that can prevent further damage to the cochlea and auditory system.

## 1. Introduction

Noise-induced hearing loss (NIHL) is a growing public health concern, particularly among younger populations due to increased exposure to loud sounds from various sources [1]. Although NIHL has historically been linked to occupational risks, the widespread use of personalized listening devices (PLDs) such as wireless earbuds, music players, and cellphones has become a significant non-occupational source of exposure to high-intensity noise. These devices, often worn for extended periods at high volume, have the potential to permanently damage cochlear hair cells, which might result in irreversible hearing loss, particularly in the high-frequency range [2].

The cochlea, a spiral-shaped organ in the inner ear, is responsible for translating sound vibrations into nerve impulses that are interpreted by the brain. Within the cochlea, hair cells are particularly sensitive to high-intensity sounds [3]. Long-term exposure to noise levels above 85 dB can damage or destroy these hair cells, resulting in hearing loss at high frequencies, typically between 3 and 8 kHz. The initial symptoms of hearing loss are often mild, such as trouble comprehending speech in noisy situations or a muffled hearing experience. As a result, individuals may wait to seek medical assistance until more serious damage has occurred [4]. A growing body of evidence suggests that the misuse of PLDs is an emerging risk factor for NIHL, especially among young adults and adolescents [5]. According to estimates from the World Health Organization (WHO), unsafe listening habits with high volume put over 1 billion young people at risk of hearing loss globally. PLDs are frequently used in noisy locations, such as crowded places or public transport, which leads users to increase the volume to dangerously high levels to block out outside noise [6]. Furthermore, PLD’s user-friendly and compact nature contribute to their prolonged usage, which can range for hours every day, increasing cumulative noise exposure to the auditory system [7].

One of the key issues with PLD-induced hearing loss is that the damage occurs gradually. High-frequency hearing loss may not manifest noticeable symptoms until significant damage has occurred [8]. First to be affected are high-frequency sounds, which are essential for comprehending speech consonants, and background noises like sirens or alarms. People may eventually start to develop more overt hearing issues, such as tinnitus (ear ringing) or trouble recognizing speech in noisy surroundings, as additional frequencies get involved. Unfortunately, once the cochlea’s hair cells are destroyed, they cannot be repaired, thus early identification and prevention are vital [9]. Despite the growing concern over NIHL related to PLDs, there is limited research specifically focused on the effects of prolonged PLD use among medical students, a population that may be at higher risk due to long study hours and frequent use of PLDs to relieve stress through music or podcasts [10,11]. Medical students often use these devices for extended periods while studying, commuting, exercising, and in noisy environments where higher volumes are required to mask external noise [12].

The present study aims to evaluate the prevalence of hearing loss caused by high-intensity noise in medical students who use PLDs. By focusing on a specific group of young people who are excessively dependent on these devices, this study hopes to provide insight into the possible long-term repercussions of PLD usage in this demographic group. To develop targeted preventative interventions, it is important to understand the prevalence and risk factors of hearing loss in this population. This will help to promote safe listening habits, raise public awareness, and early detection to prevent irreparable damage.

## 2. Materials and Methods

This cross-sectional study was conducted to assess the incidence of high-frequency noise-induced hearing loss among medical students using personalized listening devices (PLDs). Ethical approval for the study was obtained from the Institutional Ethics Committee. Informed written consent was obtained from all participants before their inclusion in the study. No minors were included in this study.

Data were collected from 100 medical graduates using a semi-structured questionnaire. The questionnaire captured various aspects such as the duration of PLD use, reasons for using the devices, volume levels, exposure to loud environments (e.g., concerts), hearing symptoms such as tinnitus, muffled voices, and instances where participants increased the volume of PLDs, particularly in noisy environments. The study was conducted over a three-month period, from 1 April 2023 to 1 August 2023.

The inclusion criteria for participants were medical students aged 20 to 30 years of age (clinical year) who used PLDs.

The exclusion criteria included individuals with known ear pathology or pre-existing conditions such as otitis externa, chronic otitis media (mucosal and squamosal), sudden sensorineural hearing loss, congenital hearing loss, inner ear disorders such as Menieres disease, or any other condition that may affect hearing. Participants with long-term exposure to ototoxic drugs such as aminoglycosides, loop diuretics, and chemotherapy drugs for ear infections or immunocompromised conditions were excluded. Participants with a history of previous ear surgeries, hearing aids, or significant medical history (including psychiatric illness and neurological illness affecting hearing) were also excluded. Additionally, students who did not use PLDs were also excluded.

Every participant had a comprehensive history taken along with an elaborate clinical examination, which included tuning fork tests to check air and bone conduction and otoscopic evaluation to rule out middle ear and external ear pathology. A conventional pure-tone audiometry was carried out for frequencies from 250 Hz to 8000 kHz, followed by high-frequency audiometry for 9000 kHz to 20,000 kHz on all subjects in a soundproof room with minimal ambient noise. Hearing at 13 distinct frequencies, spanning from 250 Hz to 20 kHz, was assessed using both pure-tone audiometry (250 Hz to 8000 kHz) and extended high-frequency audiometry (9000 kHz to 20,000 kHz).

The audiometry findings were recorded in terms of frequencies (Hz) and decibels (dB). Each participant received a comprehensive audiogram. As per the Goodman and Clarke classification, hearing loss was categorized into five categories: mild (26–40 dB), moderate (41–55 dB), moderately severe (56–70 dB), severe (71–90 dB), and profound (>91 dB) for frequencies from 250 Hz to 8000 kHz. The categorization assisted in determining the degree of hearing loss in connection to PLD usage using conventional PTA. Average hearing thresholds were further obtained for frequencies from 9000 kHz to 20,000 kHz.

Version 21 of SPSS was used for statistical analysis. Descriptive statistics, such as percentages and frequencies, were employed to summarize the data. The categorical variables were compared using the Pearson Chi-Square test. Significance was defined by a *p*-value less than 0.05 using a two-tailed test. Subsequently, the findings were examined to ascertain the frequency and severity of hearing impairment, specifically focusing on high-frequency hearing loss, which is commonly associated with extended exposure to loud noises via PLDs.

## 3. Results

A total of 100 medical students participated in the study, with an equal gender distribution of 50 males and 50 females. Based on the clinical examination and conventional pure-tone audiometry (250 Hz to 8 kHz), hearing loss was identified in 33% of the participants. Among these, 21 males (42.9%) and 12 females (23.5%) had hearing loss (Table A1).

Regarding the duration of exposure to personalized listening devices (PLDs) (Table A2), 96% of the participants reported using them for more than 4 years, while only 2% had used them for 2 to 4 years and another 2% for less than 2 years. Of those using PLDs for more than 4 years, 45% exhibited clinical symptoms such as tinnitus, voice muffling, and ear fullness. Among the students with 2 to 4 years of PLD use, one participant (50%) had reported clinical symptoms. Similarly, one of the two participants who had used PLDs for less than 2 years reported clinical symptoms.

Regarding PLD usage, Table A3 represents the relationship between PLD (personal listening device) usage settings and hearing loss among the medical students. Of the participants using PLDs in noisy environments, 53.8% reported hearing loss, while 46.2% had normal hearing. These findings suggest that exposure to high noise levels while using PLDs may increase the risk of hearing impairment among medical students.

Table A4 reveals that unilateral hearing loss affected 63.6% of the individuals, while bilateral hearing loss affected 36.4% of the participants. Right-sided hearing loss was seen in 19% of the participants, while left-sided hearing loss was observed in 28%. Among the 19% with right-sided hearing loss, most lateralization was found to occur at the 4 kHz frequency (mild hearing loss). Among the 28% with left-sided hearing loss, the maximum frequency for mild hearing loss was at 4 kHz and at 8 kHz for moderate hearing loss (Table A5).

**Figure A1:**
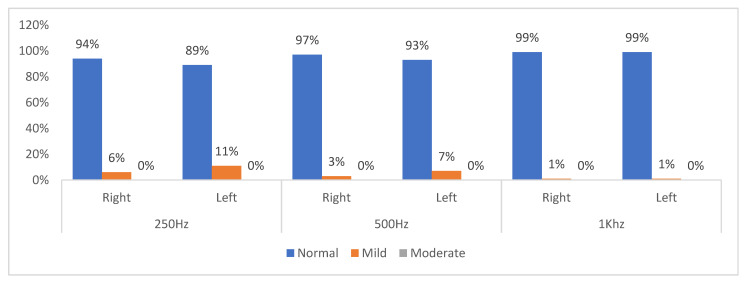
Hearing loss among medical graduates: frequency-specific analysis.

**Figure A2:**
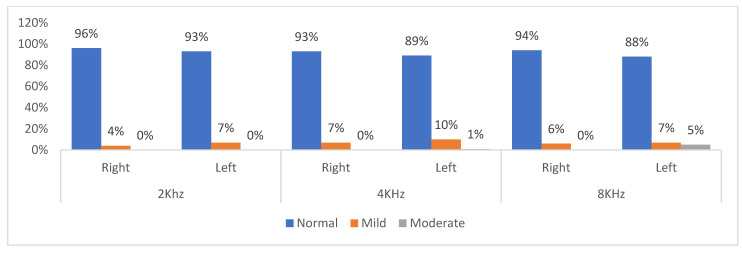
Hearing loss among medical graduates: frequency-specific analysis.

The prevalence of hearing loss across frequencies is summarized in (Figure A1 and Figure A2). Mild hearing loss was more common in the left ear (11% and 7%) compared to the right ear (6% and 3%) at lower frequencies of 250 Hz and 500 Hz, respectively. The prevalence of mild hearing loss decreased at 1000 kHz affecting only 1% of the participants in both ears. However, at higher frequencies (2000 kHz, 4000 kHz, and 8000 kHz), the prevalence of mild hearing loss increased with the left ear consistently showing higher rates (7%,10%, and 7%) than the right ear (4%,7% and 6%). Notably moderate hearing loss was observed only in the left ear at 4000 kHz (1%) and 8000 kHz (5%). These findings suggest left ear is more susceptible to both mild and moderate hearing loss, particularly at high frequencies.

In Table A6, 43% of the participants admitted to using headphones, while 57% did not use headphones at all. The analysis revealed a significant correlation between the development of symptoms and the usage of headphones. Hard of hearing was reported by 20.9% of the headphone users while 18.6% experienced tinnitus. Among non-users, 3.5% reported hard of hearing and 1.8% reported tinnitus (*p* < 0.0001).

The duration of usage of PLD per day (hours) had a significant correlation with hearing loss with *p*-value < 0.0001 (Table A7 and Figure A4).

**Figure A3:**
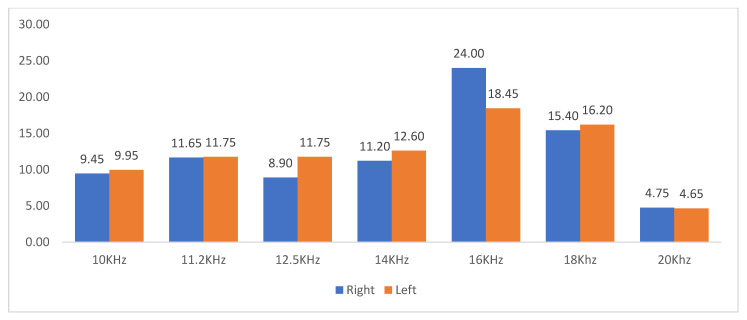
Mean hearing threshold—extended high frequency.

**Figure A4:**
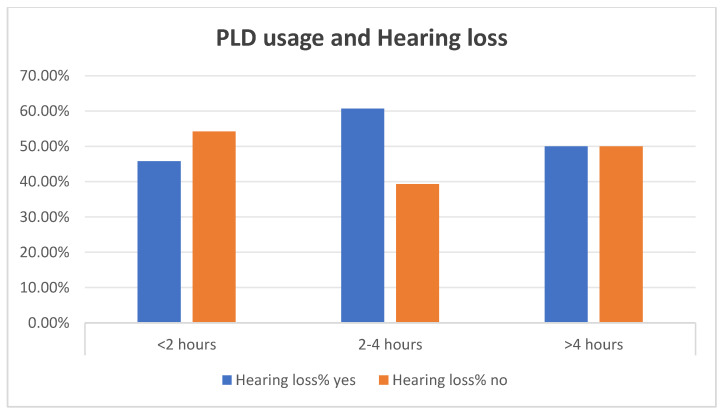
PLD usage and hearing loss.

On comparing the mean average threshold of the participants at extended high frequencies (9 kHz to 20,000 kHz), hearing thresholds were significantly elevated at 16 kHz and 18 kHz in both ears [Figure A3]. Elevated hearing threshold was generally seen in the participants who used PLD for extended periods of time.

A multivariate logistic regression model was used to examine the factors contributing to hearing loss focusing on hearing threshold 250 hz to 8000 kHz (Table A8). The analysis identified male sex as a significant predictor of hearing loss across frequencies from 250 Hz to 8 kHz, with males having 7.4 times higher odds compared to females (Exp(B) = 7.386, 95% CI: 1.629–33.496, *p* = 0.010). Other factors, such as exposure to loud noise, duration of exposure, noisy environments, headphone usage, and symptoms like tinnitus or difficulty hearing, showed no statistically significant associations.

## 4. Discussion

The findings of this study demonstrate a significant incidence of high-frequency noise-induced hearing loss (NIHL) among medical students using personalized listening devices (PLDs). This is a growing concern, particularly given the increasing prevalence of PLD use in younger populations. The fact that 33% of the participants exhibited hearing loss, predominantly affecting males, highlights the substantial impact of long-term exposure to high-intensity noise from PLDs. This aligns with the existing literature, which suggests that the frequent use of PLDs, especially at high volumes and in noisy environments, can lead to cumulative damage to the delicate structures of the cochlea, particularly at higher frequencies. Several studies have shown that exposure to noise through headphones can be responsible for the incidence of hearing loss among users. In this study, there was a strong relation between headphone usage and the presence of symptoms such as hard of hearing and tinnitus. This finding corroborates those of Haruna K et al. [13] and Keppler et al., who also observed significant hearing loss among PLD users [14].

According to our research, students who have used PLD for more than four years are more likely to experience clinical symptoms including voice muffling and tinnitus, which are the early signs of auditory impairment. This is consistent with other studies that show that extended exposure to loud noise causes progressive cochlear damage, initially manifesting as high-frequency hearing loss. The fact that symptoms are more common in people who use PLDs for shorter periods suggests that hearing loss can develop relatively quickly, even with little exposure.

Additionally, AlOmari et al. (2024) demonstrated that adults with high PLD exposure levels reported more hearing symptoms, including tinnitus, which was echoed in our study. Tinnitus and voice muffling were common symptoms among our participants, particularly those with over four years of PLD use [15]. The study by You et al. (2020) also emphasized that while students may understand the risks associated with high PLD volume, many still engage in risky habits, such as listening at high volumes in noisy environments. This discrepancy between knowledge and behavior reflects our study’s finding, where students continued unsafe listening habits despite their awareness of potential risks [16]. Our current study highlights the risk of hearing impairment among PLD users in noisy environments. However, students using PLD in noisy environments may also be exposed to various other noise sources such as urban living, social events, and other leisure activities. Future research should investigate such cumulative effects of these noise exposures on hearing loss to provide a more comprehensive understanding of the factors contributing to this growing public health concern.

The prevalence of left-sided hearing loss (28.0%) in our study was notably higher, indicating potential asymmetry in exposure, possibly linked to the ear that is preferred for using headphones. This finding may be related to individual PLD usage behavior, such as preferring one ear over the other. Ogbe et al. (2014) noted significant individual variations existing in how people use PLDs, including the tendency to prefer one ear over the other when using headphones. This lateralization can lead to uneven exposure to sound, potentially increasing the risk of hearing loss in the preferred ear. However, their results showed that the prevalence of increased hearing thresholds was 20.3% in the right ear and 18.8% in the left ear [17]. Another study by Alshamrani. R et al. among university students in Saudi Arabia found significantly lower amplitudes in the left ear in the upper quartile of the category “volume of PLD use” which corroborates with our study [18]. The higher prevalence of left-sided hearing loss in our study highlights the importance of considering individual differences and preferences in PLD usage behavior when assessing hearing loss risks. However, our study did not collect specific data on ear preference during PLD use, suggesting that further research is needed to investigate this potential relationship and explore other contributing factors.

A noteworthy percentage of pupils experienced bilateral hearing loss (36.4%), indicating that long-term exposure to high-intensity sounds can result in widespread cochlear damage. Symmetric exposure in both ears, particularly when using headphones or earphones that provide identical sound pressure, can result in the involvement of NIHL bilaterally.

Furthermore, research highlighting the exacerbating influence of background noise, which drives users to turn up the volume of their devices above safe listening limits supports the correlation between PLD usage in loud surroundings and a greater prevalence of hearing loss (53.8%). The findings of our study align closely with the growing body of research on the risks of noise-induced hearing loss (NIHL) associated with the prolonged use of personalized listening devices (PLDs). We found that 33% of the medical students had hearing loss, with a higher incidence among those using PLDs for more than four years and in noisy environments. This corresponds with the results of Mahmood et al. (2022), who reported an auditory threshold shift in 46.7% of students using PLDs, suggesting that prolonged use can significantly contribute to high-frequency hearing loss. Their study also identified that the continuous use of PLDs for more than eight years increased the risk of hearing loss, reinforcing our findings, where most participants had used PLDs for over four years with hearing loss predominantly at high frequencies [19].

According to Natarajan et al. (2023), workplace noise exposure is a major contributing factor to the multi-factorial etiology of noise-induced hearing loss (NIHL), caused by both environmental and genetic factors. The majority of hearing-impaired students (53.8%) in our study utilized PLDs in loud settings, indicating that exposure to outside noise exacerbates the negative effects of PLDs. This feature was also discussed in the Natarajan et al. study, highlighting the compounded risk of using PLDs in already noisy settings, which accelerates cochlear damage and leads to high-frequency NIHL [8].

When Li et al. (2019) looked at speech recognition in PLD users versus non-users, they discovered that PLD users had a far worse capacity to handle time-compressed speech in noisy environments. This supports our finding that students with PLDs are more susceptible to hearing impairments in loud settings, indicating that auditory processing and speech perception are important domains affected by high-frequency NIHL [20].

As humans, we can perceive sound ranging from as low as 20 Hz to as high as 20,000 kHz. Various studies have used extended high-frequency threshold assessments to monitor the early effect of noise-induced hearing loss. High-frequency hearing loss in our study was most pronounced at 16 kHz, which aligns with the work of Le Prell et al. (2022), who noted that extended high-frequency (EHF) hearing is often adversely affected by noise exposure [21]. This is further substantiated by Sulaiman et al.’s work which confirms a significant increase in mean hearing threshold among PLD users at higher frequencies [22].

Studies showing that the 16 kHz frequency range is particularly susceptible to noise-induced damage are consistent with the high prevalence of hearing loss in this range, seen in both unilateral and bilateral instances [23]. The majority of hearing loss in this range in our sample may be attributed to the degeneration of outer hair cells in the cochlea caused by exposure to high-intensity sound. Our study results, therefore, signify the importance of prioritizing auditory health among young adults.

The year of study (pre-clinical vs. clinical) may also influence medical student’s PLD usage patterns. As students progress from pre-clinical to clinical years, their academic demands or workload, along with work pressure, long hospital hours shifts, and lifestyle habits, can make them more vulnerable to PLD usage as a coping mechanism for stress. They may also further resort to increased PLD usage to acquire access to higher educational resources for further studies and specialization. Future studies should investigate these factors to gain a deeper insight into such parameters which could help develop targeted interventions and promote healthy listening practices.

In summary, our research emphasizes that long-term PLD usage carries a significant risk of NIHL, especially in noisy environments. We recommend educating medical students about the dangers of the prolonged use of personal listening devices (PLDs) and promoting safe listening practices. Incorporating noise-canceling technology in PLDs could help reduce volume levels by eliminating ambient noise and thereby listening to audio content at a much lower volume. This can indirectly minimize the risk of noise-induced hearing loss [24].

Additionally, educational campaigns that target safe listening habits should be promoted to raise awareness among the youth population. World Health Organization has launched the “Make Listening Safe” which provides guidelines for safe listening practice. This includes taking regular breaks, limiting volume to 60% of the maximum level, moving away from loud noise, and using earplugs in noisy environments (e.g., clubs) followed by regular hearing health checkups. Manufacturers and policymakers also play an important role. Manufacturers can implement volume-limiting features in PLDs and policymakers can establish set guidelines and regulations for the safe use of PLDs. By working together, we can create a safer listening environment and reduce the risk of hearing loss associated with PLD users [25].

Future research should focus on longitudinal studies to evaluate the progression of hearing loss over time and identify the potential risk factors. Additionally, intervention studies could be conducted to evaluate strategies for mitigating the risks associated with prolonged PLD usage.

Recommendation: The importance of routine hearing assessments should be emphasized in educational programs, particularly for those who spend considerable time in noisy environments. Noise-induced hearing loss can be prevented by adopting safe listening practices, such as setting volume limits and taking pauses when using PLDs. Additionally, incorporating high-frequency audiometry into routine physical examinations can help in early detection. Future research should explore protective strategies such as noise-canceling devices and the influence of earbud varieties on auditory health integrity.

Limitation: The limitations of this research work include the small sample size of 100 medical students which may not be representative of the larger population. Self-reported data on the regularity and circumstances of PLD usage pose recall bias. The participants are from a single medical college and this may pose a convenience bias. Longitudinal follow-up was excluded, which can limit our ability to track the progression of hearing loss over time. Future research should involve a larger, more diverse population, including follow-up over a longer period for a better understanding of the evolution of hearing loss.

## 5. Conclusions

The prevalence of hearing loss among young medical graduates is a growing concern. Accurate diagnosis by these populations during their clinical practice in the future is also going to rely on their ability to detect subtle sounds and findings through auscultation using a stethoscope. Therefore, their auditory health plays a critical role in their day-to-day medical practice. This study adds to the increasing amount of data showing early-onset high-frequency hearing loss in young adults as a result of long-term PLD usage, especially in loud surroundings. These results highlight the urgent need for preventative interventions, such as regular audiometry screening for at-risk populations, public health campaigns promoting safe listening practices, and the development of noise-limiting features in PLDs to protect the well-being of both healthcare provider and their patients.

## Data Availability

No new data were created, all available data are contained within.

## Appendix A

**Table A1:** Gender and hearing loss.

Gender	Total	Hearing Loss (%)	Normal (%)
Male	50	21 (42.9%)	28 (57.1%)
Female	50	12 (23.5%)	39 (76.5%)
Total	100	33 (33%)	67 (67%)

**Table A2:** Duration of PLD use and clinical symptoms.

Duration of PLD Use	Total (%)	Clinical Symptoms Present (%)	No Clinical Symptoms (%)	*p*-Value
>4 years	96 (96%)	32 (45%)	64 (55%)	0.788
2–4 years	2 (2%)	1 (50%)	1 (50%)	
<2 years	2 (2%)	1 (50%)	1 (50%)	
Total	100	34 (34%)	66 (66%)	

**Table A3:** Usage of PLD and hearing loss.

PLD Usage	Total (Out of 100)	Hearing Loss (%)	Normal (%)
Noisy Environment	13	7 (53.8%)	6 (46.2%)

**Table A4:** Unilateral and bilateral hearing loss.

Unilateral HL	21 (63.6%)
Bilateral HL	12 (36.4%)
Total	33 (33%)

**Table A5:** Lateralization of hearing loss.

Side	Hearing Loss Present (%)	Frequency with Maximum Loss	Frequency with Minimum Loss
Right	19.0%	4 kHz (mild HL)	1 kHz (mild HL)
Left	28.0%	4 kHz (mild HL) 8 kHz (moderate HL)	1 kHz (mild HL)
Both Sides	12.0%	4 kHz (most common)	

**Table A6:** Relation between headphone usage and symptoms.

		Symptoms						*p*-Value
		Hard of Hearing		Tinnitus		No		
		Count	Row N %	Count	Row N %	Count	Row N %	
Headphone usage	No	2	3.5%	1	1.8%	54	94.7%	<0.0001
	Yes	9	20.9%	8	18.6%	26	60.5%	

**Table A7:** PLD duration of exposure and hearing loss.

		Hearing Loss %	*p*-Value
Duration of exposure	<2 h	45.8%	<0.0001
	2–4 h	60.7%	
	>4 h	50.0%	

**Table A8:** Multivariate logistic regression for hearing threshold between 250 Hz and 8000 kHz.

	B	*p*-Value	Exp(B)	95% C.I.for EXP(B)	
				Lower	Upper
Sex (Male)	2.000	0.010	7.386	1.629	33.496
Exposure to loud noise (2–4 years)	−0.173	0.819	0.841	0.192	3.688
Exposure to loud noise (>4 years)	−1.119	0.333	0.327	0.034	3.140
Duration of exposure (2–4 h)	1.451	0.070	4.267	0.889	20.474
Duration of exposure (>4 h)	0.705	0.558	2.025	0.191	21.445
Noisy environment (Yes)	1.749	0.360	5.748	0.136	243.586
Headphone usage (Yes)	1.414	0.424	4.111	0.128	131.629
Symptoms (tinnitus)	0.663	0.470	1.940	0.322	11.709
Symptoms (hard of hearing)	1.756	0.136	5.792	0.574	58.396
Constant	−3.395	0.114	0.034		
